# Supplementary material for: Identification of photoperiod-induced specific miRNAs in the adrenal glands of Sunite sheep (Ovis aries)
Source: Front Vet Sci. 2022 Jul 22;9:888207. doi: 10.3389/fvets.2022.888207 (PMC9354845; doi:10.3389/fvets.2022.888207)
Supplement: Supplementary file 1 [file Data_Sheet_1.ZIP › Supplementary file/miRDeep2 file.zip!/miRDeep2 file/Novel_Structure/SP42A1/Novel_99.pdf]

Provisional ID	:	NC_019460.2_6948
Score total	:	2.3
Score for star read(s)	:	-1.3
Score for read counts	:	0
Score for mfe	:	2
Score for randfold	:	1.6
Score for cons. seed	:	
Total read count	:	66
Mature read count	:	66
Loop read count	:	0
Star read count	:	0

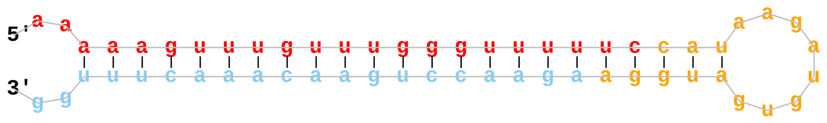

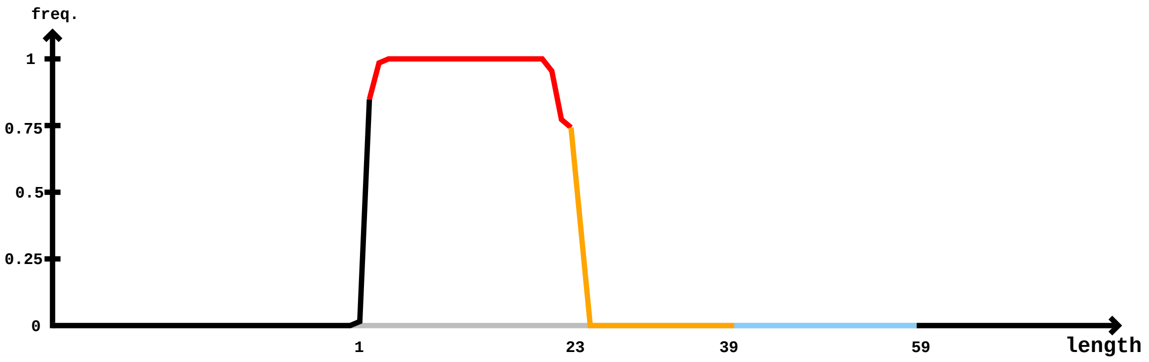

Mature Star

```
5'- acguagaggaaaccaaaauuugggguugaccaaaaaguuuguuuggguuuuuccauaagaugugauggaagaaccugaacaaacuuuggccaacccaauuaaagguuau   -3'    exp
    ..........((((........((((((.((((..(((((((((((((((((((((........))))))))))))))))))))))))).)))))).......))))..   reads    mm    sample
    ..............................Aaaaaaguuuguuuggguuu...........................................................   1        1     seq
    ...............................aaaaaguuCguuuggguuu...........................................................   2        1     seq
    ...............................aaaaaguuCguuuggguuuu..........................................................   12       1     seq
    ...............................aaaaaguuuguCuggguuuuu.........................................................   1        1     seq
    ...............................aaaaaguuuguuuggguuuuu.........................................................   1        0     seq
    ...............................aaaaaguuCguuuggguuuuuc........................................................   38       1     seq
    ...............................aaaaaguuuguuuggguuuuuU........................................................   1        1     seq
    ................................Gaaaguuuguuuggguuuuuc........................................................   1        1     seq
    ................................aaaaguuuguuuggguuuuuA........................................................   1        1     seq
    ................................aCaaguuuguuuggguuuuuc........................................................   1        1     seq
    ................................Gaaaguuuguuuggguuuuucc.......................................................   5        1     seq
    ................................aaaaguuuguuuggguuuuucc.......................................................   1        0     seq
    .................................aaaguuuguuuggguuuuuU........................................................   1        1     seq
```
